# Effect of in-hospital and post-discharge complications on 1-year functional outcome after stroke and transient ischemic attack

**DOI:** 10.1093/esj/23969873251383315

**Published:** 2026-01-01

**Authors:** Christian Boehme, Lukas Mayer-Suess, Thomas Toell, Anel Karisik, Kurt Mölgg, Silvia Felicetti, Benjamin Dejakum, Lucie Buergi, Lukas Scherer, Karin Willeit, Wilfried Lang, Johann Willeit, Peter Willeit, Michael Knoflach, Stefan Kiechl, Raimund Pechlaner

**Affiliations:** Department of Neurology, Medical University of Innsbruck, Innsbruck, Austria; Department of Neurology, Medical University of Innsbruck, Innsbruck, Austria; Department of Neurology, Medical University of Innsbruck, Innsbruck, Austria; Department of Neurology, Medical University of Innsbruck, Innsbruck, Austria; VASCage - Research Centre on Vascular Ageing and Stroke, Innsbruck, Austria; Department of Neurology, Medical University of Innsbruck, Innsbruck, Austria; VASCage - Research Centre on Vascular Ageing and Stroke, Innsbruck, Austria; Department of Neurology, Medical University of Innsbruck, Innsbruck, Austria; Department of Neurology, Medical University of Innsbruck, Innsbruck, Austria; VASCage - Research Centre on Vascular Ageing and Stroke, Innsbruck, Austria; VASCage - Research Centre on Vascular Ageing and Stroke, Innsbruck, Austria; Department of Neurology, Medical University of Innsbruck, Innsbruck, Austria; VASCage - Research Centre on Vascular Ageing and Stroke, Innsbruck, Austria; Medical Faculty, Sigmund Freud Private University, Vienna, Austria; Department of Neurology, Medical University of Innsbruck, Innsbruck, Austria; Institute of Clinical Epidemiology, Public Health, Health Economics, Medical Statistics, and Informatics, Medical University of Innsbruck, Innsbruck, Austria; Department of Public Health and Primary Care, University of Cambridge, Cambridge, UK; Ignaz Semmelweis Institute, Interuniversity Institute for Infection Research, Vienna, Austria; Department of Neurology, Medical University of Innsbruck, Innsbruck, Austria; VASCage - Research Centre on Vascular Ageing and Stroke, Innsbruck, Austria; Department of Neurology, Medical University of Innsbruck, Innsbruck, Austria; VASCage - Research Centre on Vascular Ageing and Stroke, Innsbruck, Austria; Department of Neurology, Medical University of Innsbruck, Innsbruck, Austria

**Keywords:** complications, functional outcome, population-level impact

## Abstract

**Introduction:**

Complications may secondarily impair functional outcome after stroke and transient ischemic attack (TIA). Here, we estimated the population-level impact of complications on long-term functional outcome to describe their combined impact and identify the most impactful complications.

**Patients and methods:**

Patients admitted for acute ischemic stroke or TIA (ABCD2 score ⩾ 3) and discharged without severe disability were followed-up for occurence of 12 in-hospital and 26 post-discharge complications. Population-level impact of complications on the primary endpoint of non-excellent functional outcome (modified Rankin Scale (mRS) > 1) at end of follow-up 12 months after stroke was assessed by population attributable fraction (PAF). This cohort study was performed at a single European comprehensive stroke center and nested within a randomized controlled trial of intensified post-stroke care, STROKE-CARD.

**Results:**

Among 1705 patients aged 69.4 ± 13.6 years (59.8% male), 36.8% (95% confidence interval: 34.5, 39.2) had non-excellent outcome at 12 months, 21.3% (19.4, 23.3) had unfavorable outcome (mRS > 2), and functional worsening occurred in-hospital in 2.6% (2.0, 3.6) and post-discharge in 20.3% (18.4, 22.3). Non-excellent 1-year functional outcome was predicted in-hospital by occurrence of recurrent stroke, neurological worsening, and infections (PAF: 0.6%–3.2%). Post-discharge, 12 complications significantly predicted outcome, and pain, severe fatigue, falls, and depression were most impactful (PAF: 5.4%–13.0%). Together, in-hospital complications accounted for 7.6% and post-discharge complications for 31.8% of non-excellent outcome, whereas acute therapy (thrombolysis and/or thrombectomy) and STROKE-CARD care accounted for 8.0% and 14.5% of excellent outcomes. In moderate and severe stroke (NIHSS > 5), acute therapy was the strongest predictor of excellent outcome (PAF: 29.1%). Results were consistent or stronger in patients with TIA, without prestroke disability, or in the regional catchment area, and when focusing on unfavorable outcome or worsening in mRS.

**Discussion:**

Complications were responsible for more than one third of non-excellent 1-year functional outcome with pain, severe fatigue, depression, and falls most impactful. Complications rivaled the impact of acute therapy and determined functional outcome after TIA as well as after stroke.

**Conclusion:**

Effective prevention and treatment of complications may substantially improve long-term functional outcomes after stroke and TIA.

## Graphical Abstract

**Graphical Abstract:**
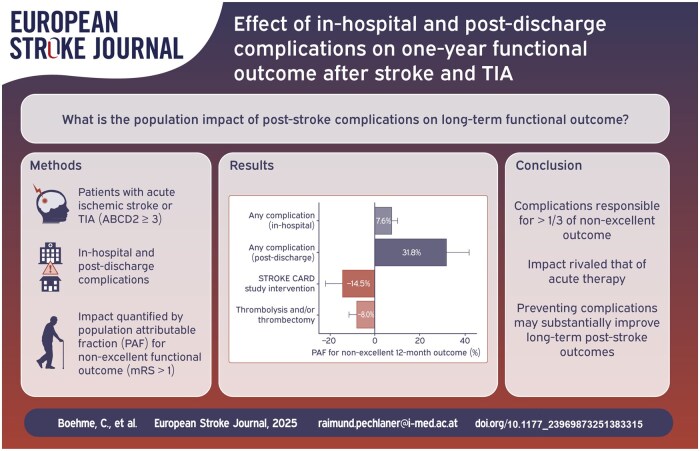

## Introduction

Stroke is a leading global cause of disability with a lifetime risk of 25%.^1–3^ Although advances in treatment including reperfusion therapy, stroke unit care, and intensified rehabilitation have considerably improved short-term outcomes, initial good outcome may be diminished by post-stroke complications. To retain achieved outcomes, long-term strategies for the management of patients after stroke are needed. Sustainable strategies specific to different settings and regions can improve life after stroke and aid detection and treatment of complications, which is a key priority of the Stroke Action Plan for Europe 2018–2030.^4,5^

Stroke-related health problems affect 90% of stroke patients after hospital discharge, 80% of which require medical interventions,^6^ and half of all stroke patients are re-hospitalized or die within 1 year.^7,8^ Recurrent cardiovascular disease including recurrent stroke, and other complications including epilepsy, fractures, fatigue, cognitive impairment, pain, and depression contribute to long-term disability, reduced quality of life, and worse functional outcome. However, intensified post-stroke care including timely prevention, detection, and treatment of complications as well as recognition of remaining rehabilitation needs can contribute to improved quality of life and functional outcome, as demonstrated by the STROKE-CARD trial.^9^

Neurological status at 24 h after thrombectomy for stroke due to anterior circulation large vessel occlusion explains approximately half of the variability in functional outcome at 3 months after stroke,^10^ suggesting the other half to be explained by other factors such as delayed neurological improvement or worsening, rehabilitation, and occurrence of complications.^10^ Preventing secondary functional deterioration may be even more powerful when baseline functional status is better, as in minor stroke and in the absence of thrombectomy.

Here, we assessed the population-level impact of accurately diagnosed in-hospital and post-discharge complications on 1-year functional outcome and compared this impact to the benefit of acute therapy and intensified post-stroke care.

## Patients and methods

### Study design

This prospective cohort study is embedded within the pragmatic open-label randomized controlled post-stroke disease management trial STROKE-CARD^9^ that investigated the effects of intensified post-stroke care on functional outcome 1 year after stroke or TIA with ABCD2 Score ⩾ 3. Intensified care included a standardized 3-month follow-up visit and a web portal to improve risk profiles, assess complications, comorbidities, and rehabilitation needs, and counsel patients, whereas both study groups received a standardized 12-month visit. Patients were recruited from January of 2014 to December of 2017. Long-term follow-up was performed up to October of 2021 (in-person) or October of 2022 (by telephone). The current analysis considered all patients recruited at the study center Innsbruck. Participants provided written informed consent and the study was approved by the ethics committee of the Medical University of Innsbruck.

### Participants and study population

Consecutive patients (*n* = 2625) aged at least 18 years with acute ischemic stroke or transient ischemic attack with an ABCD2 score ⩾ 3 who were admitted to Innsbruck University Hospital, a comprehensive stroke center, from January 2014 through December 2017 were screened. We excluded 274 patients living outside the survey area and 474 patients with conditions that interfere with cardiovascular disease and stroke prevention (Supplemental Figure 1): 60 patients with terminal illness (life expectancy < 1 year); 35 patients with documented drug or alcohol abuse; 136 patients with psychiatric disorders, severe dementia and/or inability to provide informed consent; and 243 patients who remained bedridden at discharge (modified Rankin Scale [mRS] score = 5). Of 1877 eligible patients, 1730 (92.2%) did not decline participation, and 1-year functional outcome was available for 1705, which constituted the study population (Supplemental Figure 1).

### Ascertainment of patient data

Patient data were assessed by structured interview including medical history, medical and other therapies, and post-stroke complications as well as clinical and laboratory examinations at baseline and 12 months. Further information was retrieved from medical records comprising regional hospital information systems of all hospitals in the Tyrol in addition to the national electronic health record, which records all hospitalizations, laboratory results, and imaging. Self-report was verified and complemented using these sources and direct communication with patients’ general practitioners.

### Definition of exposures

Exposures comprised carefully assessed recurrent events, procedures, and complications of ischemic stroke or TIA and are here designated *complications*. We make no claim of complications in this sense being caused directly by the index stroke; rather, they are understood as *events susceptible of intervention*, that is, adverse events occuring after stroke the prevention or better management of which may improve post-stroke outcomes. Detailed definitions of complications and other exposures are provided in the Online-only supplement. Types of complications were only considered if at least 10 participants experienced them, which resulted in 12 considered during hospital stay and 26 after discharge. Complications were recorded from the day of stroke or TIA until the 1-year follow-up visit. Acute therapy was defined as administration of intravenous thrombolysis and/or mechanical thrombectomy, and intensified post-stroke care by STROKE-CARD intervention group trial arm. Neurological worsening was defined as an increase in NIHSS of 2 points or more for each observation period individually. This choice of cut-off follows prior studies that used a 2-point increase in NIHSS as a measure of neurological deterioration^11^ that may be both sensitive and robust to some variance in NIHSS assessment.

Minor stroke was defined as stroke with admission NIHSS ⩽ 5. Catchment area was recorded as the city or district of Innsbruck versus other, distinguishing patients from the local catchment area from patients referred for treatment at a comprehensive stroke center. Stroke unit care describes patients being treated at the stroke unit during hospitalization for the index stroke or TIA.

### Definition of outcomes

Functional status at 1-year after stroke was assessed by mRS. The primary endpoint was non-excellent outcome, defined as mRS > 1. Secondary end-points included unfavorable outcome, defined as mRS > 2, and mRS worsening. Two distinct mRS worsening outcomes were defined as any increase in mRS in-hospital or post-discharge, respectively.

Valence terms of “less than excellent” and “less than good” have been proposed for mRS > 1 and mRS > 2 outcome categories.^12^ We here use “non-excellent” and “unfavorable” for these categories with the aim of expressing similar valence with greater brevity.

The joint impact of complications was estimated by creating an indicator variable reflecting the within-subject occurence of any complication of those significantly associated with the primary end-point.

Population-level impact was assessed as population attributable fraction (PAF). PAF is the proportion of adverse outcome attributable to each complication, that is, the proportion of adverse outcome that could be prevented by completely preventing the complication, under assumption of causality. PAF provides a population-level view of impact by integrating both how commonly a complication occurs in the population and how serious for each individual affected it is. As such, it is particularly useful for health economic analyses and public health priority setting. PAF could be calculated from incidences of outcomes overall and in those not affected by a specific complication in the setting of the current study by calculating (Incidence_Overall_ – Incidence_Not affected by complication_)/Incidence_Overall_, although we use a multivariable calculation method to control for potential confounding as described below.

Rating of mRS was performed by independent raters blinded to trial arm certified by the BlueCloudX training and certification platform (HealthCarePoint, Cedar Park, USA).

### Statistical analysis

Baseline characteristics are shown as mean ± standard deviation, median (interquartile range), or count (proportion). Associations of complications with functional outcomes were estimated using logistic regression including multivariable adjustment for age, sex, STROKE-CARD trial arm, NIHSS at admission, pre-stroke mRS, and stroke unit care when investigating in-hospital complications; and for age, sex, trial arm, pre-stroke and discharge mRS, and discharge NIHSS, when investigating post-discharge complications. Population attributable fractions (PAFs) were calculated using the same models and a doubly robust estimator.^13^ Joint PAFs were defined as the PAFs associated with occurrence of any of the complications significantly associated with non-excellent outcome during each observation period individually (in-hospital or post-discharge). PAFs for increasing numbers of complications occurring in the same patient were calculated by cumulatively summing PAFs for individual effects of complication counts. Predictors of length of hospital stay were examined using linear regression, adjusting for age and sex. Effect modification of associations of complications with the primary endpoint by subgroups according to age (median split into ⩾73 years vs <73 years), sex (male vs female), index event type (TIA vs minor stroke vs moderate/severe stroke), pre-existing disability (prestroke mRS 0 vs >0), and catchment area (regional vs extended) was investigated by interaction terms and analysis of deviance of nested models, with control of multiplicity by the Benjamini-Hochberg procedure.

Analysis was conducted using R version 4.3.3 (The R Foundation for Statistical Computing, Vienna, Austria).

## Results

### Characteristics of the study population

Of 1730 patients enrolled, 1-year functional outcome was available for 1705 (98.6%), which constituted the study population (Supplemental Figure 1). Patients were on average 69.4 ± 13.6 (mean ± standard deviation) years old and 59.8% were male (Table 1). Average length of hospital stay was 9.9 ± 6.2 days, of post-discharge follow-up, 369.5 ± 24.5 days, and 3.7% died before 1-year. Average mRS pre-stroke/TIA, at admission, discharge, and 1-year was 0.3, 2.3, 1.4, and 1.4. Overall, 36.8% (95% confidence interval: 34.5, 39.2) had non-excellent 1-year functional outcome, 21.3% (95% CI: 19.4, 23.3) unfavorable outcome, and mRS worsening occurred in-hospital in 2.6% (95% CI: 2.0, 3.6) and post-discharge in 20.3% (95% CI: 18.4, 22.3). In patients with TIA, corresponding proportions were 24.6% (95% CI: 19.9, 29.9), 11.8% (95% CI: 8.5, 16.1), 3.0% (95% CI: 1.4, 5.7), and 26.6% (95% CI: 21.8, 32.0).

**Table 1.** Characteristics of the study population.

Variable	Level	All	Males	Females
*n*		1705 (100.0)	1020 (59.8)	685 (40.2)
Age		69.4 ± 13.6	68.5 ± 13.3	70.8 ± 14.0
Index event	TIA	305 (17.9)	174 (17.1)	131 (19.1)
	Minor stroke	1029 (60.4)	639 (62.6)	390 (56.9)
	Moderate/severe stroke	371 (21.8)	207 (20.3)	164 (23.9)
Cerebrovascular history		391 (22.9)	250 (24.5)	141 (20.6)
Days of hospital stay		9.9 ± 6.2	9.7 ± 6.3	10.2 ± 6.1
Days of post-discharge follow-up		369.5 ± 24.5	368.6 ± 25.6	370.7 ± 22.8
Attended 12-month follow-up		1643 (96.4)	983 (96.4)	660 (96.4)
Death between discharge and 12 months		63 (3.7)	37 (3.6)	26 (3.8)
Post stroke care (STROKE-CARD trial arm)	Standard	563 (33.0)	335 (32.8)	228 (33.3)
	Intensified	1142 (67.0)	685 (67.2)	457 (66.7)
NIHSS (admission)		2.0 (4.0)	2.0 (4.0)	2.0 (4.0)
NIHSS (admission), stroke patients		3.0 (5.0)	3.0 (4.0)	3.0 (5.0)
NIHSS (discharge)		1.0 (2.0)	0.0 (2.0)	1.0 (2.0)
NIHSS (12-months)		0.0 (1.0)	0.0 (1.0)	0.0 (1.0)
mRS (prestroke)		0.0 (0.0)	0.0 (0.0)	0.0 (0.0)
mRS (admission)		2.0 (2.0)	2.0 (2.0)	2.0 (3.0)
mRS (discharge)		1.0 (2.0)	1.0 (2.0)	2.0 (2.0)
mRS (12-months)		1.0 (2.0)	1.0 (2.0)	1.0 (3.0)
MMSE (in-hospital)		27.0 (4.0)	27.0 (4.0)	28.0 (4.2)
MoCA (in-hospital)		22.1 ± 5.2	22.3 ± 5.0	21.9 ± 5.6
HADS-anxiety (in-hospital)		5.0 ± 3.6	4.6 ± 3.3	5.5 ± 3.8
HADS-depression (in-hospital)		4.0 ± 3.4	3.8 ± 3.4	4.2 ± 3.3
OABSS (in-hospital)		3.7 ± 3.1	3.4 ± 2.9	4.1 ± 3.2
FSS (in-hospital)		3.2 ± 1.5	3.0 ± 1.5	3.4 ± 1.6
Arterial hypertension (pre-existing)		1384 (81.2)	828 (81.2)	556 (81.2)
Diabetes (pre-existing)		311 (18.2)	211 (20.7)	100 (14.6)
Dyslipidemia (pre-existing)		1461 (85.7)	883 (86.6)	578 (84.4)
Atrial fibrillation (pre-existing)		421 (24.7)	237 (23.2)	184 (26.9)
Epilepsy (pre-existing)		43 (2.5)	24 (2.4)	19 (2.8)
Regional catchment area		1188 (69.7)	689 (67.5)	499 (72.8)
Thrombectomy		64 (3.8)	34 (3.3)	30 (4.4)
Thrombolysis		273 (16.0)	161 (15.8)	112 (16.4)
Thrombolysis and/or thrombectomy		286 (16.8)	171 (16.8)	115 (16.8)
TOAST	Cardioembolism	434 (25.5)	248 (24.3)	186 (27.2)
	Large artery atherosclerosis	356 (20.9)	241 (23.6)	115 (16.8)
	Small artery occlusion	356 (20.9)	208 (20.4)	148 (21.6)
	Stroke of other determined cause	75 (4.4)	39 (3.8)	36 (5.3)
	Stroke of undetermined cause	484 (28.4)	284 (27.8)	200 (29.2)

TIA: transient ischemic attack; NIHSS: National Institutes of Health Stroke Scale; mRS: modified Rankin Scale; HADS: hospital anxiety, and depression scale; MMSE: mini-mental status exam; MoCA: Montreal Cognitive Assessment; OABSS: Overactive Bladder Symptom Score; FSS: Fatigue severity score; TOAST: Trial of ORG 10172 in Acute Stroke Treatment.

Baseline characteristics were assessed during hospital stay for the index cerebrovascular event (stroke or TIA). Characteristics are shown as mean ± standard deviation, median (interquartile range), or count (proportion).

### Complication rates

There were 12 complications occurring during hospitalization and 26 between discharge and 1-year for which at least 10 events were registered (Supplemental Table 1). At least one complication occurred in 16.1% (95% CI: 14.4, 18.0) in-hospital and in 89.9% (95% CI: 88.2, 91.3) post-discharge. At least one of the complications individually associated with non-excellent outcome occurred in 12.6% (95% CI: 11.1, 14.3) and 81.1% (95% CI: 79.1, 82.9). Supplemental Table 3 provides an overview of participant characteristics by complication occurence.

### Primary outcome analysis

**Figure 1.**
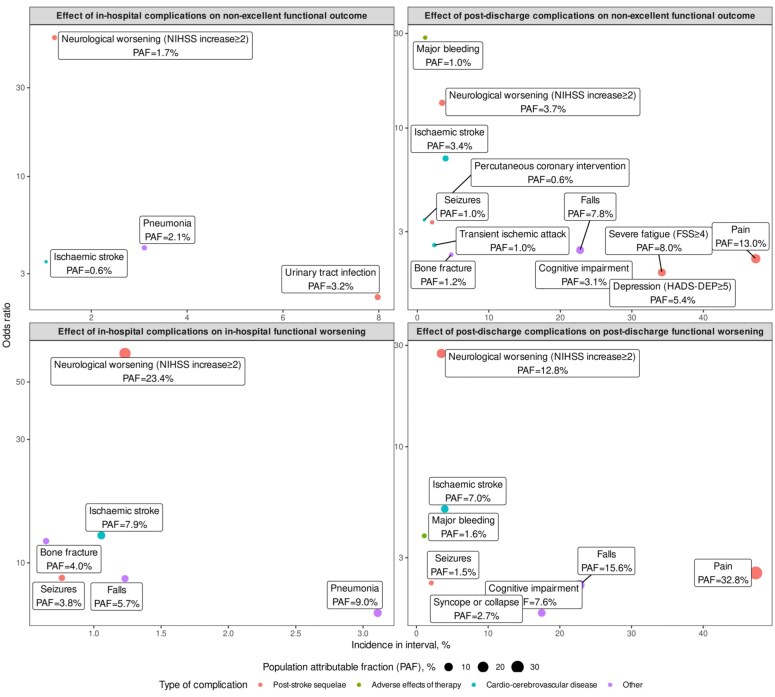
Population-level and individual-level impact of post-stroke complications on non-excellent outcome and on worsening in functional outcome. Complications that were significantly associated with non-excellent outcome are shown by their period incidence (*x* axis) and individual-level odds ratio for functional outcomes (*y* axis), for non-excellent outcome or mRS worsening for in-hospital and post-discharge periods (panels). Population-level impact (PAF, population attributable fraction) is provided in labels. PAF: Population attributable fraction; NIHSS: National Institutes of Health Stroke Scale.

Figure 1 shows how population impact is driven by individual-level association with outcome (y axis) and period incidence (x axis) for individual complications, and Supplemental Table 2 provides more detail. Whereas during hospitalization, infections including pneumonia und urinary tract infections had greatest impact on non-excellent 1-year outcome, after discharge pain, severe fatigue, falls, and depression were most impactful. Compared to these complications, neurological worsening, recurrent stroke, and major bleeding occurred less frequently and had lesser impact.

Together, in-hospital complications accounted for 7.6% (95% CI: 5.2, 10.0) and post-discharge complications for 31.8% (95% CI: 21.5, 42.0) of non-excellent 1-year functional outcome. For comparison, attributable fractions of acute therapy (thrombolysis and/or thrombectomy) and of intensified post stroke care (STROKE-CARD study intervention) were -8.0% (95% CI: −11.4, −4.7) and −14.5% (95% CI: −22.0, −7.0), respectively.

### Subgroup analyses

**Figure 2.**
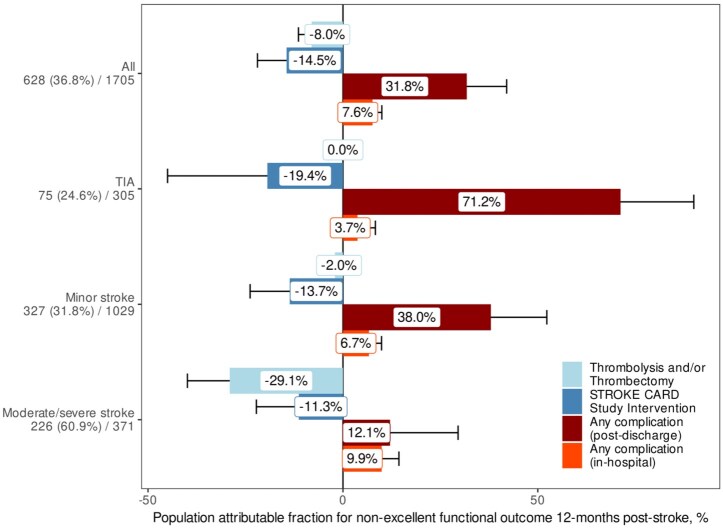
Population-level impact of complications, acute therapy, and intensified post-stroke care, overall and by stroke severity. Bar length and labels indicate population-level impact (PAF) in terms of non-excellent 1-year functional outcome (mRS > 1) of occurrence of any complication in-hospital or post-discharge, respectively, of acute therapy (thrombolysis and/or thrombectomy), and of the STROKE-CARD post-stroke disease management intervention. Error bars indicate 95% confidence intervals. Numbers in y axis labels indicate count (percentage) of patients with non-excellent outcome and group size for each group. Whereas in severe stroke, acute therapy and in-hospital complications had the greatest impact on non-excellent functional outcome, in TIA and minor stroke post-discharge complications and intensified post-stroke care were most important. PAF, Population attributable fraction; mRS, modified Rankin Scale; TIA, transient ischemic attack; NIHSS, National Institutes of Health Stroke Scale.

Stroke severity was inversely related to impact of post-dicharge complications on the primary outcome (PAFs: TIA 71.2%, mild stroke 38.0%, moderate/severe stroke 12.1%). Conversely, more severe stroke resulted in higher impact of in-hospital complications (PAFs: TIA 3.7%, mild stroke 6.7%, moderate/severe stroke 9.9%) and of acute therapy (PAFs: TIA 0.0%, mild stroke −2.0%, moderate/severe stroke −29.1%). (Figure 2). Similar findings emerged for individual complications (Supplemental Figure 2a). Apart from type of index event/stroke severity, other subgroups in which complications had greater impact included younger patients, men, and those without pre-existing disability (Supplemental Figures 2c and d).

### Impact of multiple complications

**Figure 3.**
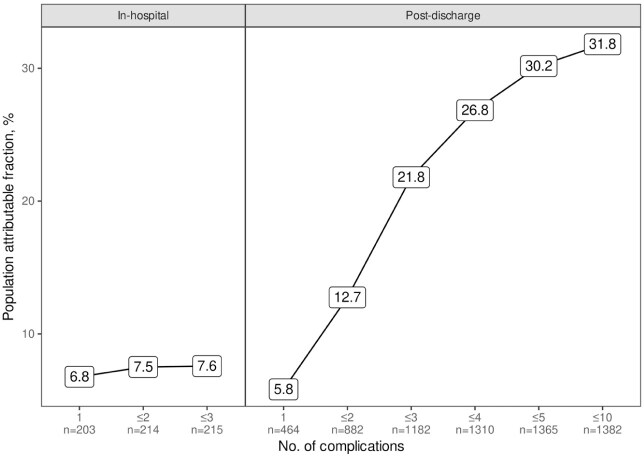
Population-level impact of occurrence of multiple complications. Numbers give cumulative population attributable fractions of non-excellent outcome due to multiple complications occurring in the same patient, with complication count and cumulative numbers of patients that suffered any and up to as many complications given as *x*-axis labels. During hospital stay, multiple complications and one single complication similarly impacted outcome; few patients suffered multiple complications. Post-discharge, increasing number of complications had increasing impact on outcome up to occurrence of five complications; most patients suffered multiple complications. PAF: Population attributable fraction; TIA: transient ischemic attack; NIHSS: National Institutes of Health Stroke Scale.

Outcome worsened when multiple post-discharge complications occurred, with each additional complication in the same patient increasing odds of non-excellent outcome by a factor of 1.64 (95% CI: 1.48, 1.83) with corresponding increases in population impact (Figure 3). During hospital stay, patients rarely experienced multiple complications and population-level impact was driven by occurrence of any complication.

### Falls

Falls occurred in-hospital in 1.23% (0.79, 1.91) and post-discharge in 22.8% (20.8, 24.9; Supplemental Table 2). Compared to patients that did not fall, patients with 1, 2, or 3 or more falls after discharge had 1.87 (95% CI: 1.33, 2.62), 4.17 (95% CI: 1.75, 10.42), and 6.42 (95% CI: 1.39, 35.67) higher odds of non-excellent outcome, respectively.

### Secondary outcomes

Results were similar but not identical for the primary and secondary outcomes. Unfavorable outcome (mRS > 2) or mRS worsening was not predicted by post-discharge occurrence of severe fatigue, depression, or fractures (Supplemental Figure 3).

### Length of hospital stay

In-hospital complications prolonged hospital stay, with recurrent stroke and epileptic seizures entailing the longest excess stay (Table 2). Occurrence of any complication prolonged hospital stay by 5.3 (95% CI: 4.5, 6.2) days.

**Table 2.** Effect of in-hospital complications on length of hospital stay.

Complication	Excess length of stay, days (95% CI)	*p*-Value
Recurrent stroke	12.5 (9.7, 15.3)	<0.001
Seizures	11.8 (8.5, 15.1)	<0.001
Neurological worsening (NIHSS increase ⩾ 2)	9.2 (6.6, 11.8)	<0.001
Major bleeding	8.8 (4.9, 12.6)	<0.001
Transient ischemic attack	7.7 (4.3, 11.0)	<0.001
Pneumonia	6.4 (4.8, 8.1)	<0.001
Any complication	5.3 (4.5, 6.2)	<0.001
Myocardial infarction	4.7 (1.0, 8.3)	0.013
Falls	4.5 (1.9, 7.2)	<0.001
Bone fracture	4.5 (0.8, 8.2)	0.016
Heart failure	4.3 (0.8, 7.8)	0.017
Urinary tract infection	3.1 (2.0, 4.2)	<0.001
Deep vein thrombosis	1.5 (−2.4, 5.3)	0.450

Complications occurring during hospitalization were generally associated with significantly longer hospital stay. Recurrent ischemic stroke and epileptic seizures had the largest effect on length of stay, whereas occurrence of any complication prolonged hospital stay by approximately 5 days. Average length of stay was 9.9 ± 6.2 days.

NIHSS: National Institutes of Health Stroke Scale.

## Discussion

This prospective analysis highlights that the cumulative burden of complications following acute ischemic stroke or TIA is a major determinant of 1-year functional outcome.

Approximately 90% of patients suffered one or more complications, most of which occurred post-discharge, in line with prior reports.^6^ In-hospital and post-discharge complications were responsible for 7.6% and 31.8% of non-excellent outcome (Figure 2), whereas acute therapy (thrombolysis and/or thrombectomy) and STROKE-CARD care favored excellent outcome (PAF: −8.0% and −14.5%). In moderate and severe stroke (NIHSS > 5), acute therapy was the strongest predictor of excellent outcome (PAF: −29.1%). STROKE-CARD care showed beneficial effects in TIA, minor stroke, and moderate/severe stroke (Figure 2) and focused on better rehabilitation, secondary prevention, and recognition of common complications. Most strokes were minor strokes (mean NIHSS, 4.3), which corresponds to a well-described secular trend of decreasing stroke severity^14^ attributed to improved recognition and better risk factor management in primary prevention,^15^ including atrial fibrillation detection and treatment.^8,16^

Population-level impact was largest in younger patients, men, those without pre-existing disability, and patients with TIA (Supplemental Figure 2). These findings suggest defined groups of patients in which intensified prevention of complications may yield most benefit.

Post-discharge pain, severe fatigue, falls, and depression occurred in 20%–40% of patients, which is similar as previously reported^17–22^ and resulted in the largest impact on functional outcome. By comparison, recurrent stroke, neurological deterioration, and treatment side effects such as major bleeding were more serious at the individual level, but because they were much less common, had smaller impact at the population level (Figure 1 and Supplemental Table 2). Post-stroke depression impairs functional outcome and rehabilitation.^23,24^ Chronic pain impairs quality of life and associates with spasticity, depression, fatigue, anxiety, impaired physical activity, and cognitive decline.^25^

Falls were common and impactful (Figure 1 and Supplemental Table 2) with in particular multiple falls in the same patient showing strong associations with non-excellent functional outcome. Falls occurred post-discharge in 22.8%, and proportions as high as 40% have been reported.^26,27^ Stroke entails a threefold increased fall risk partly due to gait, mobility, balance, visual, mood, and cognitive impairments, and to vertigo and medication effects.^28–30^ Interventions can reduce the risk of falls by around 23%.^28^ Approximately 20% of falls result in fractures or head injury,^29^ which aligns closely with our results (Supplemental Table 2). Substantially increased fracture risk after stroke or TIA has been reported, with predisposing factors of more severe comorbidity, higher age, and female sex.^31^ Fractures result in a large health burden and reduced quality of life.^31^ Here, we report for the first time the high population-level impact of fractures on non-excellent functional outcome after stroke, calling for commensurate incorporation of fall and fracture prevention in post-stroke care.

Stroke is associated with a twofold elevated risk of dementia, and post-stroke dementia entails significant excess mortality.^32,33^ Impact of post-stroke cognitive impairment was moderate (PAF: 3.1%) in this study sample excluding patients with pre-existing psychiatric illness, diagnosis of dementia, or severe stroke (discharge mRS ⩾ 5). Interestingly, population impact of cognitive impairment was largest in patients with TIA (Supplemental Figure 2a), in agreement with recent reports of faster cognitive decline after TIA.^34^

Our results on in-hospital complications align with prior reports that identified falls, pneumonia, and urinary tract infections as most common.^35–41^ Period incidences found here are lower than previously reported,^35^ possibly explained by shorter hospital stay, improved stroke and post-stroke care, and high proportions of mild stroke. Occurrence of in-hospital complications prolonged length of hospital stay by approximately 5 days, although more severe complications including recurrent stroke, neurological worsening, major bleeding, and epileptic seizures resulted in an approximate doubling of length of stay (Table 2). These results reflect economic impact of complications, as in-hospital care is the most expensive part of stroke care.^42^

Secondary analyses focused on population-level impact of complications on any worsening in mRS and on unfavorable functional outcome (mRS > 2; Supplemental Figure 3). mRS worsening was associated with additional complications including in-hospital falls, fractures, and seizures, suggesting greater population impact when considering any worsening. Unfavorable outcome (mRS > 2) was predicted by fewer, more severe complications, whereas fatigue, depression, and fractures associated exclusively with the primary endpoint. Such differences are consistent with qualitative aspects of mRS, as these complications, although barring patients from performing all previous activities (mRS = 1), may often permit the ability to look after own affairs without assistance (mRS = 2).

Our results can be considered conservative because we excluded very severe stroke (mRS = 5) and dementia, which may reduce the impact of post-stroke epilepsy, cognitive impairment, and other complications. Moreover, some types of complications were not considered, such as sleep disturbance, spasticity, and delirium. The STROKE-CARD study intervention may have resulted in lower complication impact by improved recognition of some complications, including depression, anxiety, spasticity, or cognitive impairment, but not others, including falls, fractures, or fatigue. One further limitation pertains to the observational study design, which cannot define the causal relationship between complications and outcome. However, we comprehensively adjusted for functional and neurological status at the beginning of each observation period, and results were stronger when restricting analysis to patients without pre-existing disability (Supplemental Figure 2d). Advancements in secondary stroke prevention made after recruitment of study patients are not reflected in current results, which may particularly affect results on cardiovascular complications including stroke and myocardial infarction. Although analysis was embedded within a randomized intervention trial, analyses were adjusted for trial arm and the effect of the trial intervention is shown (Figure 2). Findings are based on a homogeneous study population of overwhelmingly Caucasian descent in an economically developed country and may not necessarily generalize to different populations.

Strengths include high enrollment of 92.2% of eligible patients and rigorous and highly complete follow-up of 98.6% (Supplemental Figure 1). Complications were assessed using validated instruments and criteria and utilizing multiple comprehensive national and local electronic health record systems. Complications had similar impact in patients residing in the regional catchment area and patients referred for access to a comprehensive stroke center (Supplemental Figure 2e).

## Conclusion

Complications accounted for more than one third of non-excellent 1-year functional outcome after stroke and TIA. Their impact was greatest post-discharge, in TIA and minor stroke, in younger patients, men, and those without pre-existing disability. Pain, severe fatigue, depression, and falls were most common and impactful. Major improvements in functional outcome may be attainable by focused prevention, detection, and treatment of post-stroke and TIA complications.

## Supplementary Material

ds-eso_23969873251383315

## Trial registration

The STROKE-CARD trial was registered at ClinicalTrials.org (registration number: NCT02156778).

## The STROKE-CARD Study Group

Markus Anliker, Christian Boehme, Gregor Broessner, Julia Ferrari, Martin Furtner, Julian Granna, Andrea Griesmacher, Ton Hanel, Viktoria Hasibeder, Katharina Kaltseis, Stefan Kiechl, Gerhard Klingenschmid, Michael Knoflach, Theresa Koehler, Stefan Krebs, Florian Krismer, Clemens Lang, Wilfried Lang, Lukas Mayer-Suess, Christoph Mueller, Wolfgang Nachbauer, Anna Neuner, Raimund Pechlaner, Anja Perfler, Thomas Porpaczy, Gerhard Rumpold, Christoph Schmidauer, Gudrun Schoenherr, Lisa Seekircher, Martin Sojer, Christine Span, Lydia Thiemann, Thomas Toell, Lena Tschiderer, Marlies Wichtl, Johann Willeit, Karin Willeit, Peter Willeit.

## Data availability

Anonymized patient data may be made available for use in independent scientific research to researchers upon reasonable request to the last author pending approval of a research proposal, including a statistical analysis plan, and completion of a data sharing agreement.
