# Supplementary material for: Effect of in-hospital and post-discharge complications on 1-year functional outcome after stroke and transient ischemic attack
Source: Eur Stroke J. 2026 Jan 1;11(1):23969873251383315. doi: 10.1093/esj/23969873251383315 (PMC12866259; doi:10.1093/esj/23969873251383315)
Supplement: ds-eso_23969873251383315 [file ds-eso_23969873251383315.zip › sj-docx-1-eso-10.1177_23969873251383315.docx]

Supplement to:

**Effect of in-hospital and post-discharge complications on one-year functional outcome after stroke and transient ischemic attack**

Christian Boehme^1^, Lukas Mayer-Suess^1^, Thomas Toell^1^, Anel Karisik^1,2^, Kurt Mölgg^1,2^, Silvia Komarek^1^, Benjamin Dejakum^1^, Lucie Buergi^2^, Lukas Scherer^2^, Wilfried Lang^3^, Johann Willeit^1^, Peter Willeit^4,5,6^, Michael Knoflach^1,2^, Stefan Kiechl^1,2^, Raimund Pechlaner^1^, on behalf of the STROKE-CARD Study Group

^1^Department of Neurology, Medical University of Innsbruck, Austria.

^2^VASCage - Research Centre on Vascular Ageing and Stroke, Innsbruck, Austria.

^3^Department of Neurology, St. John's Hospital, Vienna, Austria.

^4^Institute of Clinical Epidemiology, Public Health, Health Economics, Medical Statistics, and Informatics, Medical University of Innsbruck, Innsbruck, Austria.

^5^Department of Public Health and Primary Care, University of Cambridge, Cambridge, United Kingdom.

^6^Ignaz Semmelweis Institute, Interuniversity Institute for Infection Research, Vienna, Austria.

[Supplementary Methods 3](#__RefHeading___Toc4615_1828352315)

[Ascertainment of post-stroke complications 3](#__RefHeading___Toc4617_1828352315)

[Definitions of post-stroke complications 5](#__RefHeading___Toc4619_1828352315)

[Supplemental Tables 7](#__RefHeading___Toc4621_1828352315)

[Supplemental Table 1: Complication overview. 7](#__RefHeading___Toc4623_1828352315)

[Supplemental Table 2: Population-level impact of post-stroke complications on functional outcome. 12](#__RefHeading___Toc4625_1828352315)

[Supplemental Table 3: Characteristics of participants by complication occurrence. 16](#__RefHeading___Toc4627_1828352315)

[Supplemental Figures 19](#__RefHeading___Toc4629_1828352315)

[Supplemental Figure 1. Study flow chart. 19](#__RefHeading___Toc4631_1828352315)

[Supplemental Figure 2. Impact of complications in subgroups. 20](#__RefHeading___Toc4633_1828352315)

[Supplemental Figure 3. Impact of post-stroke complications on secondary endpoints. 26](#__RefHeading___Toc4635_1828352315)

[References 27](#__RefHeading___Toc4637_1828352315)

# Supplementary Methods

## Ascertainment of post-stroke complications

Complications that occurred after the index stroke or transient ischemic attack and before a 12-month post-stroke follow-up assessment or death were analyzed according to occurence either during hospital stay or post-discharge. Participants consented to the study’s full access to medical records, which were gathered from relevant hospitals, specialists in private practice, and general practitioners. All post-stroke complications were gathered and validated using the Austrian electronic health record (ELGA) and the local hospital electronic health record (KIS).

Cardiovascular outcomes were ascertained by an outcome adjudication committee blinded to study group assignment based on critical review of patient records, imaging, electrocardiograms, and laboratory reports using diagnostic criteria listed below.^1^^,2^ Disagreements of committee members were decided by majority vote.

Neurological worsening was defined as an increase in NIHSS (National Institutes of Health Stroke Scale) of at least 2 points during an observation period (in-hospital or post-discharge). Fractures were defined as any fracture diagnosed by a radiologist based on imaging, irrespective of fracture location or whether there was a single or multiple fractures. Fractures directly caused by acute stroke/TIA were not considered. Cardiovascular procedures including cardiac pacemaker implantation, coronary artery bypass surgery, heart valve surgery, percutaneous coronary intervention, and peripheral vascular bypass were recorded by self-report and verified using medical records. Other complications included epileptic seizures (recorded by structured interview and electronic health records) and falls (recorded by structured interview).

Adverse states were assessed using validated instruments, including overactive bladder (Overactive Bladder Symptom Score ≥7 points), cognitive impairment (Montreal Cognitive Assessment ≤17 points or Mini Mental State Examination [MMSE] ≤23), anxiety (Hospital Anxiety and Depression Scale Anxiety subscale [HADS-ANX] ≥5), depression (Hospital Anxiety and Depression Scale Depression subscale [HADS-DEP] ≥5), fatigue (Fatigue Severity Scale ≥4) and pain and discomfort (EQ-5D-3L subdomain ≥1 points). These were coded as present during the post-discharge period if at least one of the in-hospital and 12-month assessments indicated pathology, based on the assumption of their relative stability and to reduce the impact of transient short-term post-stroke alterations.

Infectious disease outcomes included pneumonia and urinary tract infection both during hospitalization and after discharge. These were recorded by structured interview, laboratory results and chest radiographs and confirmation from national and local electronic medical records. Urinary tract infections after discharge were only considered if they resulted in hospitalization.

For each observation period (in- hospital, post-discharge) and the primary end-point of mRS≥2 at 12 months, compound variables reflecting occurrence of any of the complications individually significantly associated with functional outcome were created to estimate of joint PAFs for occurrence of any complication.

Complete case analysis was performed to manage missingness in exposure and outcome variables. A total of 19 missing values in the adjustment variable prestroke mRS were median-imputed.

Definitions of post-stroke complications

- Ischemic stroke^1^ was defined as an episode of neurological dysfunction caused by focal cerebral, spinal, or retinal infarction, based on (i) pathological, imaging, or other objective evidence of cerebral, spinal cord, or retinal focal ischaemic injury in a defined vascular distribution; or (ii) clinical evidence of cerebral, spinal cord, or retinal focal ischemic injury based on symptoms persisting ≥24 hours or until death, and other etiologies excluded.
- Transient ischemic attack was defined as transient focal neurological deficit with a duration of less than 24 hours and without evidence of ischemic lesion on diffusion-weighted MRI.
- Hemorrhagic stroke^1^ was defined as rapidly developing clinical signs of neurological dysfunction attributable to a focal collection of blood within the brain parenchyma or ventricular system that is not caused by trauma.
- Myocardial infarction^2^ was defined as acute myocardial injury with clinical evidence of acute myocardial ischemia and with detection of a rise and/or fall of cardiac troponin values with at least one value above the 99th percentile upper reference limit and at least one of the following: (i) symptoms of myocardial ischemia, (ii) new ischaemic electrocardiographic changes, (iii) development of pathological Q waves, (iv) imaging evidence of new loss of viable myocardium or new regional wall motion abnormality in a pattern consistent with an ischaemic aetiology, or (v) identification of a coronary thrombus by angiography or autopsy (not for type 2 or 3 myocardial infarction).
- Angina pectoris was classified as meeting two of three of the following characteristics: (i) constricting discomfort in the front of the chest or in the neck, jaw, shoulder, or arm; (ii) precipitated by physical exertion; (iii) relieved by rest or nitrates within 5 min.^3^
- Heart failure was defined as reduced left ventricular ejection fraction (LVEF) ≤50%, or those with symptoms and signs of heart failure, with evidence of structural and/or functional cardiac abnormalities and/or raised natriuretic peptides (NPs), and with an LVEF ≥50%.^4^
- Deep veinous thrombosis was defined as blood clot in the deep veins of the extremities or pelvis diagnosed by ultrasound or magnetic resonance venography.^5^
- Major bleeding was defined as fatal bleeding and/or symptomatic bleeding in a critical area or organ, such as intracranial, intraspinal, intraocular, retroperitoneal, intra-articular or pericardial, or intramuscular with compartment syndrome, and/or bleeding causing a fall in haemoglobin level of ≥20 g/L, or leading to transfusion of ≥2 units of whole blood or red cells.^6^
- Syncope was defined as transient loss of consciousness due to cerebral hypoperfusion, characterized by a rapid onset, short duration, and spontaneous complete recovery.^7^
- Peripheral artery disease was defined as symptomatic or asymptomatic atherosclerotic narrowing of arterial blood vessels in the lower extremities diagnosed according to ankle-brachial-index.^8^

# Supplemental Tables

## **Supplemental Table 1**: Complication overview.

| **Complication** | **n (%)** |
| --- | --- |
| **In-hospital** |  |
| Angina pectoris | 1 (0.1%) |
| Aortic aneurysm | 0 |
| Aortic aneurysma stenting | 0 |
| Aortic aneurysma surgery | 0 |
| Bone fracture* | 11 (0.6%) |
| Cardiac pacemaker | 6 (0.4%) |
| Cardiovascular surgery | 1 (0.1%) |
| Coronary artery bypass surgery | 0 |
| Deep vein thrombosis* | 10 (0.6%) |
| Falls* | 21 (1.2%) |
| Haemorrhagic stroke | 3 (0.2%) |
| Heart failure* | 12 (0.7%) |
| Heart valve surgery | 0 |
| Ischaemic stroke* | 18 (1.1%) |
| Major bleeding* | 10 (0.6%) |
| Myocardial infarction* | 11 (0.6%) |
| Neurological worsening (NIHSS increase≥2)* | 21 (1.2%) |
| New arterial hypertension | 0 |
| New atrial fibrillation | 3 (0.2%) |
| New diabetes | 0 |
| New dyslipidemia | 0 |
| Other cardiovascular disease | 1 (0.1%) |
| Percutaneous coronary intervention | 0 |
| Peripheral vascular bypass | 0 |
| Peripheral vascular disease | 0 |
| Peripheral vascular stenting | 0 |
| Pneumonia* | 53 (3.1%) |
| Pulmonary embolism | 2 (0.1%) |
| Rhabdomyolysis | 1 (0.1%) |
| Seizures* | 13 (0.8%) |
| Subdural haemorrhage | 1 (0.1%) |
| Sudden cardiac arrest | 3 (0.2%) |
| Syncope or collapse | 3 (0.2%) |
| Transient ischemic attack* | 13 (0.8%) |
| Urinary tract infection* | 136 (8.0%) |
| **Post-discharge** |  |
| Angina pectoris* | 12 (0.7%) |
| Anxiety (HADS-ANX≥5)* | 749 (43.9%) |
| Aortic aneurysm | 1 (0.1%) |
| Aortic aneurysma stenting | 2 (0.1%) |
| Aortic aneurysma surgery | 1 (0.1%) |
| Bone fracture* | 78 (4.6%) |
| Cardiac pacemaker* | 18 (1.1%) |
| Cardiovascular surgery | 2 (0.1%) |
| Cognitive impairment* | 298 (17.5%) |
| Coronary artery bypass surgery | 8 (0.5%) |
| Deep vein thrombosis | 5 (0.3%) |
| Depression (HADS-DEP≥5)* | 733 (43.0%) |
| Falls* | 374 (21.9%) |
| Haemorrhagic stroke | 5 (0.3%) |
| Heart failure* | 25 (1.5%) |
| Heart valve surgery* | 11 (0.6%) |
| Ischaemic stroke* | 65 (3.8%) |
| Major bleeding* | 18 (1.1%) |
| Myocardial infarction* | 13 (0.8%) |
| Neurological worsening (NIHSS increase≥2)* | 51 (3.0%) |
| New arterial hypertension* | 98 (5.7%) |
| New atrial fibrillation* | 43 (2.5%) |
| New diabetes* | 18 (1.1%) |
| New dyslipidemia* | 48 (2.8%) |
| Other cardiovascular disease | 0 |
| Overactive bladder (OABSS≥7)* | 373 (21.9%) |
| Pain* | 807 (47.3%) |
| Percutaneous coronary intervention* | 16 (0.9%) |
| Peripheral vascular bypass | 7 (0.4%) |
| Peripheral vascular disease* | 15 (0.9%) |
| Peripheral vascular stenting | 5 (0.3%) |
| Pneumonia* | 15 (0.9%) |
| Pulmonary embolism | 5 (0.3%) |
| Rhabdomyolysis | 4 (0.2%) |
| Seizures* | 34 (2.0%) |
| Severe fatigue (FSS≥4)* | 583 (34.2%) |
| Subdural haemorrhage | 5 (0.3%) |
| Sudden cardiac arrest | 1 (0.1%) |
| Syncope or collapse* | 68 (4.0%) |
| Transient ischemic attack* | 39 (2.3%) |
| Urinary tract infection | 8 (0.5%) |

Shown are number of events (percentage of study population) for each complication initially considered. Only complications for which at least 10 events during an observation period were recorded were included in analysis (*), which resulted in 12 in-hospital and 26 post-discharge complications included out of 41 distinct complications considered. Only newly registered or diagnosed complications were considered, except for anxiety, cognitive impairment, depression, overactive bladder, pain, and severe fatigue, which were deemed present after discharge if detected using standardized assessments either during hospital stay or at the 12-month follow-up. Cognitive impairment was defined as MoCA≤17 or MMSE≤23.

FSS, fatigue severity scale; HADS, hospital anxiety and depression scale; NIHSS, National Institutes of Health Stroke Scale; OABSS, overactive bladder symptom score; mRS, modified Rankin Scale; MMSE, mini-mental status exam; MoCA, Montreal Cognitive Assessment;

## **Supplemental Table 2**: Population-level impact of post-stroke complications on functional outcome.

|  |  | **Non-excellent outcome (12-months)** | | **Unfavorable outcome (12-months)** | | **mRS worsening** | |
| --- | --- | --- | --- | --- | --- | --- | --- |
| **In-hospital complications** | **Incidence** | **OR** | **PAF** | **OR** | **PAF** | **OR** | **PAF** |
| Any | 12.61 (11.09, 14.30) | 3.44 (2.41, 4.92) | 7.59 (5.21, 9.97) | 3.90 (2.73, 5.56) | 13.88 (9.60, 18.17) | 8.57 (4.35, 16.76) | 36.41 (20.89, 51.93) |
| Urinary tract infection | 7.98 (6.76, 9.39) | 2.25 (1.47, 3.45) | 3.22 (1.52, 4.92) | 3.31 (2.17, 5.07) | 7.90 (4.62, 11.19) | 1.02 (0.24, 3.02) | 0.14 (-7.54, 7.82) |
| Pneumonia | 3.11 (2.36, 4.08) | 4.14 (2.06, 8.83) | 2.06 (0.92, 3.21) | 2.99 (1.57, 5.68) | 2.96 (0.78, 5.14) | 6.41 (2.00, 17.42) | 9.04 (0.02, 18.06) |
| Neurological worsening | 1.23 (0.78, 1.91) | 55.79 (10.59, 1032.47) | 1.74 (0.82, 2.66) | 16.92 (5.78, 54.89) | 2.70 (1.16, 4.24) | 64.44 (23.40, 187.44) | 23.42 (10.97, 35.86) |
| Ischaemic stroke | 1.06 (0.65, 1.70) | 3.48 (1.13, 10.80) | 0.59 (0.02, 1.16) | 2.98 (0.87, 9.41) | 0.74 (-0.20, 1.69) | 12.77 (3.32, 40.77) | 7.94 (-0.21, 16.10) |
| Falls | 1.23 (0.78, 1.91) | 2.33 (0.81, 7.06) | 0.48 (-0.14, 1.09) | 2.18 (0.77, 6.09) | 0.78 (-0.26, 1.82) | 8.70 (1.85, 30.55) | 5.68 (-1.49, 12.86) |
| Heart failure | 0.70 (0.38, 1.26) | 3.58 (0.90, 17.67) | 0.42 (-0.05, 0.89) | 3.76 (0.95, 14.97) | 0.74 (-0.27, 1.75) | 2.83 (0.15, 16.84) | 1.37 (-2.98, 5.72) |
| Myocardial infarction | 0.65 (0.34, 1.19) | 2.95 (0.73, 15.62) | 0.35 (-0.24, 0.94) | 1.33 (0.32, 4.95) | 0.16 (-0.79, 1.11) | 2.39 (0.12, 14.70) | 1.20 (-2.73, 5.14) |
| Transient ischemic attack | 0.76 (0.42, 1.34) | 2.39 (0.64, 8.82) | 0.31 (-0.17, 0.79) | 1.84 (0.43, 6.73) | 0.33 (-0.41, 1.08) | <0.01 | -1.37 (-2.28, -0.46) |
| Seizures | 0.76 (0.42, 1.34) | 2.24 (0.60, 8.23) | 0.29 (-0.16, 0.75) | 3.44 (0.80, 12.64) | 0.60 (-0.18, 1.38) | 8.75 (1.27, 37.16) | 3.82 (-2.04, 9.67) |
| Deep vein thrombosis | 0.59 (0.30, 1.11) | 1.71 (0.39, 7.10) | 0.16 (-0.23, 0.55) | 0.88 (0.10, 4.55) | -0.04 (-0.83, 0.75) | <0.01 | -0.29 (-0.55, -0.03) |
| Major bleeding | 0.59 (0.30, 1.11) | 0.90 (0.19, 4.25) | -0.03 (-0.54, 0.49) | 1.63 (0.33, 7.65) | 0.22 (-0.52, 0.95) | <0.01 | -0.31 (-0.64, 0.01) |
| Bone fracture | 0.65 (0.34, 1.19) | 0.36 (0.05, 1.68) | -0.27 (-0.77, 0.24) | 0.55 (0.06, 2.92) | -0.21 (-0.94, 0.52) | 12.13 (1.69, 56.30) | 3.96 (-1.99, 9.92) |
| **Post-discharge complications** | **Incidence** | **OR** | **PAF** | **OR** | **PAF** | **OR** | **PAF** |
| Any | 81.06 (79.10, 82.87) | 3.22 (2.09, 5.08) | 31.77 (21.53, 42.01) | 2.34 (1.38, 4.13) | 31.52 (14.53, 48.51) | 3.14 (2.07, 4.95) | 55.04 (39.60, 70.49) |
| Pain | 47.33 (44.94, 49.73) | 2.19 (1.67, 2.89) | 13.02 (8.48, 17.57) | 2.38 (1.75, 3.28) | 20.24 (13.00, 27.47) | 2.54 (1.97, 3.31) | 32.81 (23.99, 41.64) |
| Severe fatigue (FSS≥4) | 34.19 (31.95, 36.51) | 1.87 (1.42, 2.47) | 7.99 (4.30, 11.68) | 1.08 (0.79, 1.47) | 1.29 (-4.20, 6.78) | 1.16 (0.89, 1.50) | 3.72 (-2.96, 10.40) |
| Falls | 22.76 (20.77, 24.88) | 2.42 (1.75, 3.35) | 7.81 (4.77, 10.86) | 2.73 (1.93, 3.87) | 16.02 (9.94, 22.09) | 2.24 (1.65, 3.03) | 15.63 (9.24, 22.02) |
| Depression (HADS-DEP≥5) | 42.99 (40.63, 45.38) | 1.40 (1.07, 1.84) | 5.44 (1.13, 9.76) | 0.91 (0.67, 1.24) | -1.96 (-8.49, 4.58) | 0.94 (0.73, 1.20) | -2.03 (-10.00, 5.95) |
| Neurological worsening | 3.47 (2.62, 4.57) | 13.41 (6.11, 30.58) | 3.75 (2.13, 5.36) | 19.93 (9.05, 44.85) | 7.98 (4.74, 11.22) | 27.50 (13.36, 60.75) | 12.81 (8.80, 16.83) |
| Ischaemic stroke | 3.96 (3.09, 5.04) | 7.03 (3.71, 13.53) | 3.38 (1.91, 4.85) | 6.17 (3.12, 12.10) | 4.59 (2.08, 7.09) | 5.09 (2.97, 8.74) | 6.97 (3.83, 10.10) |
| Cognitive impairment | 17.48 (15.72, 19.38) | 1.59 (1.12, 2.25) | 3.06 (0.64, 5.47) | 1.46 (1.03, 2.07) | 4.42 (0.24, 8.60) | 1.65 (1.21, 2.24) | 7.59 (2.69, 12.49) |
| Overactive bladder (OABSS≥7) | 21.88 (19.95, 23.93) | 1.20 (0.87, 1.64) | 1.46 (-1.20, 4.11) | 0.94 (0.66, 1.33) | -0.69 (-4.86, 3.49) | 0.86 (0.64, 1.15) | -2.62 (-7.80, 2.56) |
| Bone fracture | 4.75 (3.79, 5.92) | 2.30 (1.14, 4.66) | 1.22 (0.22, 2.23) | 1.76 (0.94, 3.30) | 2.03 (-0.23, 4.29) | 1.55 (0.86, 2.67) | 1.83 (-0.79, 4.46) |
| Major bleeding | 1.10 (0.67, 1.76) | 28.58 (5.40, 228.27) | 1.03 (0.28, 1.77) | 6.96 (2.07, 25.25) | 1.77 (0.24, 3.29) | 3.81 (1.36, 10.29) | 1.59 (0.17, 3.02) |
| Seizures | 2.07 (1.46, 2.91) | 3.35 (1.32, 8.89) | 0.98 (0.25, 1.72) | 2.12 (0.85, 5.13) | 1.18 (-0.57, 2.93) | 2.28 (0.97, 5.00) | 1.53 (-0.29, 3.34) |
| Transient ischemic attack | 2.37 (1.72, 3.26) | 2.57 (1.12, 5.79) | 0.95 (-0.00, 1.91) | 1.27 (0.43, 3.34) | 0.30 (-1.01, 1.60) | 1.38 (0.62, 2.84) | 0.73 (-1.06, 2.52) |
| Syncope or collapse | 4.14 (3.25, 5.25) | 1.47 (0.77, 2.77) | 0.68 (-0.60, 1.95) | 1.92 (0.97, 3.69) | 1.88 (-0.20, 3.96) | 1.94 (1.09, 3.37) | 2.69 (0.04, 5.35) |
| New atrial fibrillation | 2.62 (1.92, 3.54) | 1.79 (0.79, 4.00) | 0.61 (-0.27, 1.50) | 1.25 (0.51, 2.92) | 0.38 (-1.02, 1.78) | 1.08 (0.47, 2.25) | 0.18 (-1.62, 1.97) |
| Percutaneous coronary intervention | 0.97 (0.58, 1.61) | 3.44 (1.04, 11.65) | 0.58 (-0.07, 1.24) | 3.52 (0.88, 11.83) | 0.80 (-0.32, 1.92) | 1.59 (0.42, 4.88) | 0.40 (-0.79, 1.59) |
| Heart failure | 1.52 (1.01, 2.27) | 2.33 (0.83, 6.51) | 0.55 (-0.19, 1.30) | 1.28 (0.39, 3.82) | 0.25 (-1.07, 1.56) | 0.88 (0.28, 2.30) | -0.17 (-1.53, 1.19) |
| Myocardial infarction | 0.79 (0.44, 1.39) | 3.67 (0.95, 16.07) | 0.50 (-0.05, 1.05) | 3.91 (0.98, 14.54) | 0.89 (-0.13, 1.90) | 2.59 (0.66, 8.67) | 0.69 (-0.46, 1.85) |
| Cardiac pacemaker | 1.10 (0.67, 1.76) | 1.51 (0.47, 4.66) | 0.22 (-0.44, 0.87) | 0.82 (0.16, 3.18) | -0.12 (-0.92, 0.67) | 1.94 (0.60, 5.36) | 0.69 (-0.64, 2.02) |
| Heart valve surgery | 0.67 (0.35, 1.23) | 2.02 (0.36, 10.29) | 0.17 (-0.41, 0.74) | 1.56 (0.22, 8.59) | 0.17 (-0.88, 1.22) | 1.51 (0.31, 5.70) | 0.26 (-0.75, 1.28) |
| Pneumonia | 0.91 (0.53, 1.54) | 1.51 (0.40, 5.67) | 0.16 (-0.28, 0.61) | 2.60 (0.67, 9.25) | 0.67 (-0.43, 1.78) | 0.69 (0.11, 2.66) | -0.24 (-1.07, 0.59) |
| Peripheral vascular disease | 0.91 (0.53, 1.54) | 1.47 (0.38, 5.06) | 0.16 (-0.28, 0.60) | 0.63 (0.06, 3.32) | -0.18 (-0.82, 0.45) | 1.88 (0.56, 5.65) | 0.61 (-0.56, 1.77) |
| Angina pectoris | 0.73 (0.40, 1.31) | 1.39 (0.23, 8.02) | 0.07 (-0.35, 0.48) | 6.42 (1.16, 34.27) | 0.83 (-0.11, 1.76) | 1.59 (0.34, 5.64) | 0.31 (-0.61, 1.22) |
| New diabetes | 1.08 (0.66, 1.74) | 1.13 (0.32, 3.51) | 0.06 (-0.44, 0.55) | 1.53 (0.35, 5.50) | 0.26 (-0.44, 0.96) | 1.14 (0.31, 3.41) | 0.12 (-0.80, 1.05) |
| Anxiety (HADS-ANX≥5) | 43.93 (41.56, 46.33) | 1.00 (0.76, 1.31) | -0.05 (-4.11, 4.02) | 0.78 (0.57, 1.07) | -4.40 (-9.95, 1.15) | 0.85 (0.66, 1.10) | -4.93 (-12.59, 2.73) |
| New arterial hypertension | 5.76 (4.73, 7.01) | 0.93 (0.46, 1.80) | -0.11 (-1.08, 0.87) | 0.63 (0.22, 1.56) | -0.62 (-1.76, 0.51) | 1.24 (0.67, 2.20) | 0.73 (-1.35, 2.81) |
| New dyslipidemia | 2.84 (2.12, 3.77) | 0.79 (0.31, 1.89) | -0.19 (-0.94, 0.56) | 1.47 (0.54, 3.74) | 0.45 (-0.56, 1.46) | 1.16 (0.52, 2.37) | 0.30 (-1.33, 1.94) |

Whereas infections, neurological worsening, recurrent stroke, and falls occuring during hospital stay had greatest impact on non-excellent functional outcome at 12 months (mRS>1), pain, severe fatigue, falls, depression, and neurological worsening had greatest impact post-discharge. Cognitive impairment was defined as MoCA≤17 or MMSE≤23. Neurological worsening was defined as an NIHSS increase ≥ 2 points.

MRS, modified Rankin Scale; OR, odds ratio; PAF, population attributable fraction; INC, incidence; FSS, fatigue severity scale; HADS, hospital anxiety and depression scale; NIHSS, National Institutes of Health Stroke Scale; OABSS, overactive bladder symptom score; mRS, modified Rankin Scale; MMSE, mini-mental status exam; MoCA, Montreal Cognitive Assessment; 12M, 12 month follow-up.

## **Supplemental Table 3**: Characteristics of participants by complication occurrence.

|  |  | **In-hospital complications** | | **Post-discharge complications** | |
| --- | --- | --- | --- | --- | --- |
| **Variable** | **Level** | **None** | **Any** | **None** | **Any** |
| n |  | 1490 (87.4) | 215 (12.6) | 323 (18.9) | 1382 (81.1) |
| Age |  | 68.6 ± 13.7 | 74.7 ± 11.8 | 64.5 ± 14.5 | 70.5 ± 13.2 |
| Male sex |  | 916 (61.5) | 104 (48.4) | 210 (65.0) | 810 (58.6) |
| Index event | TIA | 290 (19.5) | 15 (7.0) | 46 (14.2) | 259 (18.7) |
|  | Minor stroke | 918 (61.6) | 111 (51.6) | 228 (70.6) | 801 (58.0) |
|  | Moderate/severe stroke | 282 (18.9) | 89 (41.4) | 49 (15.2) | 322 (23.3) |
| Cerebrovascular history |  | 326 (21.9) | 65 (30.2) | 54 (16.7) | 337 (24.4) |
| Days of hospital stay |  | 9.2 ± 5.2 | 14.5 ± 9.4 | 8.4 ± 4.4 | 10.2 ± 6.5 |
| Days of post-discharge follow-up |  | 370.1 ± 24.2 | 364.8 ± 26.5 | 371.6 ± 22.4 | 368.9 ± 25.0 |
| Attended 12-month follow-up |  | 1441 (96.7) | 202 (94.0) | 320 (99.1) | 1323 (95.7) |
| Death between discharge and 12 months |  | 50 (3.4) | 13 (6.0) | 3 (0.9) | 60 (4.3) |
| Post stroke care (STROKE-CARD trial arm) | Standard | 480 (32.2) | 83 (38.6) | 96 (29.7) | 467 (33.8) |
|  | Intensified | 1010 (67.8) | 132 (61.4) | 227 (70.3) | 915 (66.2) |
| NIHSS (admission) |  | 2.0 (3.0) | 4.0 (6.5) | 2.0 (3.0) | 2.0 (4.0) |
| NIHSS (admission), stroke patients |  | 3.0 (4.0) | 5.0 (7.0) | 2.0 (3.0) | 3.0 (5.0) |
| NIHSS (discharge) |  | 0.0 (2.0) | 2.0 (4.0) | 0.0 (1.0) | 1.0 (2.0) |
| NIHSS (12-months) |  | 0.0 (1.0) | 1.0 (3.0) | 0.0 (0.0) | 0.0 (1.0) |
| mRS (prestroke) |  | 0.0 (0.0) | 0.0 (0.0) | 0.0 (0.0) | 0.0 (0.0) |
| mRS (admission) |  | 2.0 (2.0) | 3.0 (2.0) | 2.0 (2.0) | 2.0 (2.0) |
| mRS (discharge) |  | 1.0 (2.0) | 3.0 (1.0) | 1.0 (2.0) | 2.0 (2.0) |
| mRS (12-months) |  | 1.0 (2.0) | 3.0 (3.0) | 0.0 (1.0) | 1.0 (2.0) |
| MMSE (in-hospital) |  | 28.0 (4.0) | 25.0 (6.0) | 29.0 (1.0) | 27.0 (5.0) |
| MoCA (in-hospital) |  | 22.4 ± 5.1 | 19.7 ± 5.9 | 25.1 ± 3.0 | 21.6 ± 5.4 |
| HADS-Anxiety (in-hospital) |  | 5.0 ± 3.6 | 4.9 ± 3.6 | 3.6 ± 2.8 | 5.3 ± 3.6 |
| HADS-Depression (in-hospital) |  | 4.0 ± 3.4 | 4.1 ± 3.6 | 1.5 ± 1.3 | 4.6 ± 3.4 |
| OABSS (in-hospital) |  | 3.7 ± 3.0 | 3.8 ± 3.3 | 2.8 ± 2.8 | 3.9 ± 3.1 |
| FSS (in-hospital) |  | 3.2 ± 1.5 | 3.3 ± 1.6 | 2.1 ± 0.8 | 3.4 ± 1.6 |
| Arterial hypertension (pre-existing) |  | 1190 (79.9) | 194 (90.2) | 246 (76.2) | 1138 (82.3) |
| Diabetes (pre-existing) |  | 266 (17.9) | 45 (20.9) | 42 (13.0) | 269 (19.5) |
| Dyslipidemia (pre-existing) |  | 1283 (86.1) | 178 (82.8) | 277 (85.8) | 1184 (85.7) |
| Atrial fibrillation (pre-existing) |  | 337 (22.6) | 84 (39.1) | 54 (16.7) | 367 (26.6) |
| Epilepsy (pre-existing) |  | 40 (2.7) | 3 (1.4) | 4 (1.2) | 39 (2.8) |
| Regional catchment area |  | 1037 (69.6) | 151 (70.2) | 217 (67.2) | 971 (70.3) |
| Thrombectomy |  | 47 (3.2) | 17 (7.9) | 12 (3.7) | 52 (3.8) |
| Thrombolysis |  | 221 (14.8) | 52 (24.2) | 46 (14.2) | 227 (16.4) |
| Thrombolysis and/or thrombectomy |  | 230 (15.4) | 56 (26.0) | 52 (16.1) | 234 (16.9) |
| TOAST | Cardioembolism | 350 (23.5) | 84 (39.1) | 58 (18.0) | 376 (27.2) |
|  | Large artery atherosclerosis | 313 (21.0) | 43 (20.0) | 68 (21.1) | 288 (20.8) |
|  | Small artery occlusion | 326 (21.9) | 30 (14.0) | 79 (24.5) | 277 (20.0) |
|  | Stroke of other determined cause | 69 (4.6) | 6 (2.8) | 18 (5.6) | 57 (4.1) |
|  | Stroke of undetermined cause | 432 (29.0) | 52 (24.2) | 100 (31.0) | 384 (27.8) |

Baseline characteristics were assessed during hospital stay for the index cerebrovascular event (stroke or TIA). Characteristics are shown as mean ± standard deviation, median (interquartile range), or count (proportion). Any complication refers to occurence of any of the complications individually significantly associated with the primary outcome during the respective observation period (in-hospital vs. post-discharge).

TIA, transient ischemic attack; NIHSS, National Institutes of Health Stroke Scale; mRS, modified Rankin Scale; HADS, hospital anxiety and depression scale; MMSE, mini-mental status exam; MoCA, Montreal Cognitive Assessment; OABSS, Overactive Bladder Symptom Score; FSS, Fatigue severity score; TOAST, Trial of ORG 10172 in Acute Stroke Treatment.

# Supplemental Figures

## **Supplemental Figure 1.** Study flow chart.


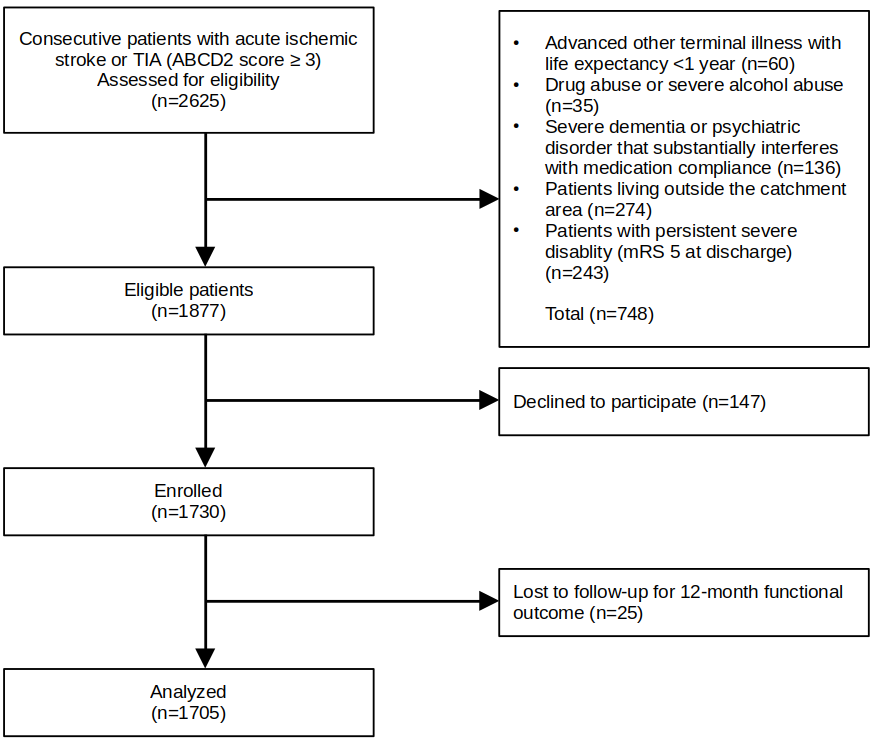


TIA: transient ischemic attack. mRS: modified Rankin Scale.

## **Supplemental Figure 2**. Impact of complications in subgroups.

Population attributable fractions for the primary endpoint of non-excellent outcome (mRS>1) are shown for complications in subgroups. Only complications significantly associated with outcome in at least one subgroup are shown, for each subplot individually.

Circles indicate significant associations, diamonds non-significant associations. Whereas there was no significant interaction of individual-level associations by subgroup membership, population-level impact was largest in younger patients, men, those without pre-existing disability, and in patients with TIA. Complications had similar impact irrespective of whether patients were admitted from the regional catchment area, or from the extended catchment area for access to a comprehensive stroke center.

*TIA, transient ischemic attack.*

***Supplemental Figure 2a*** *- Impact of post-stroke complications in subgroups by index event.*

*
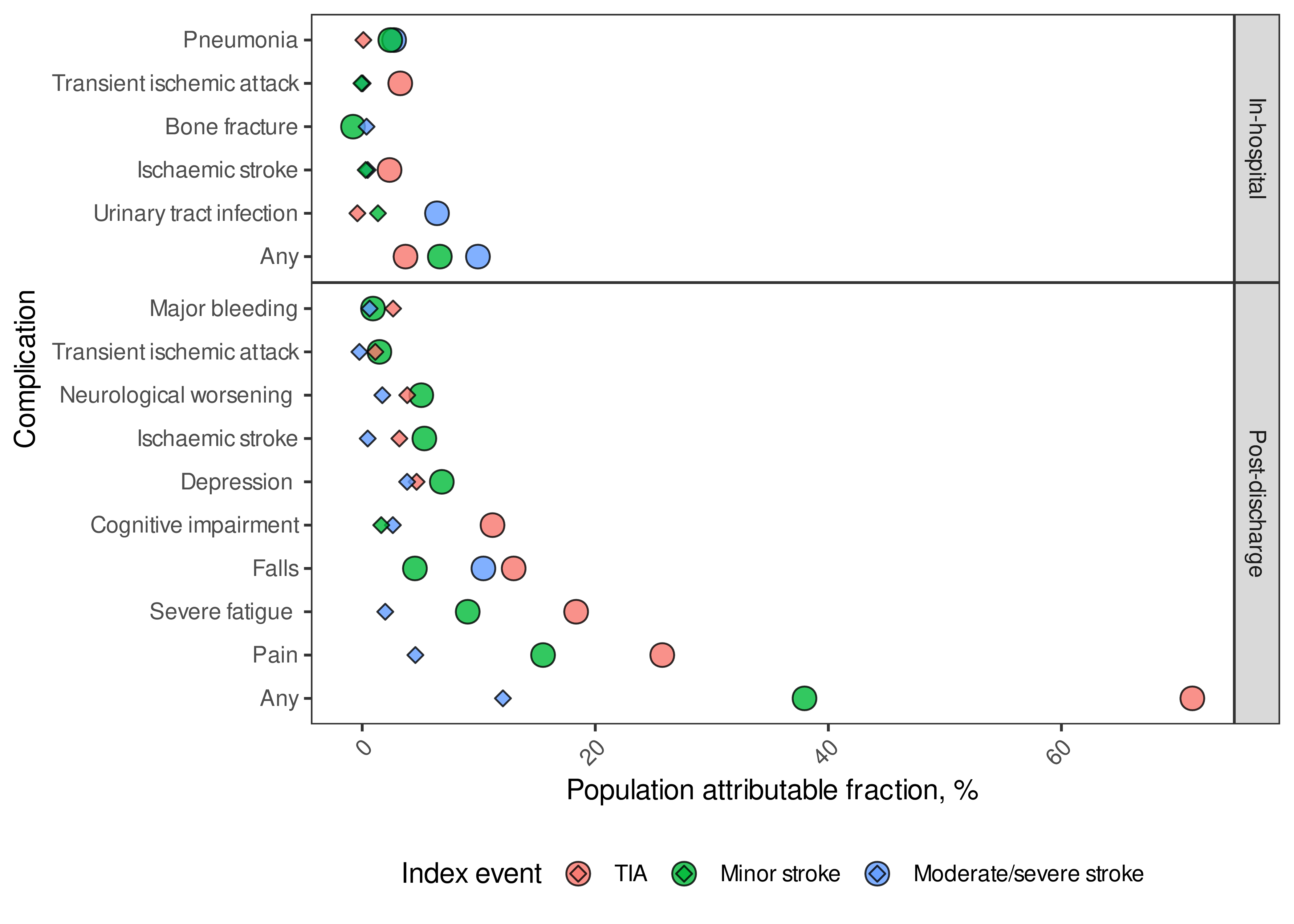
*

***Supplemental Figure 2b*** *- Impact of post-stroke complications in subgroups by age.*


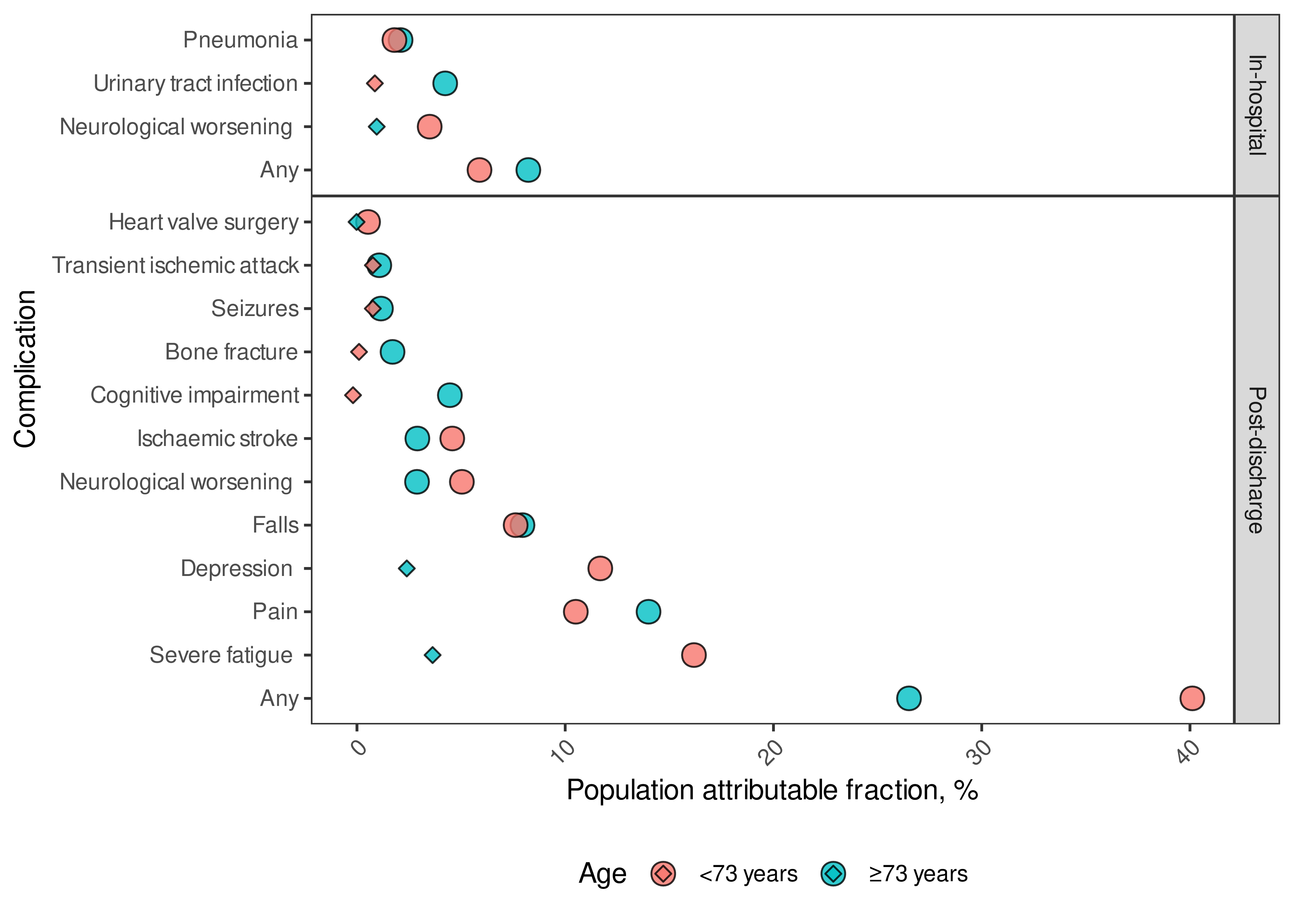


***Supplemental Figure 2c*** *- Impact of post-stroke complications in subgroups by sex.*


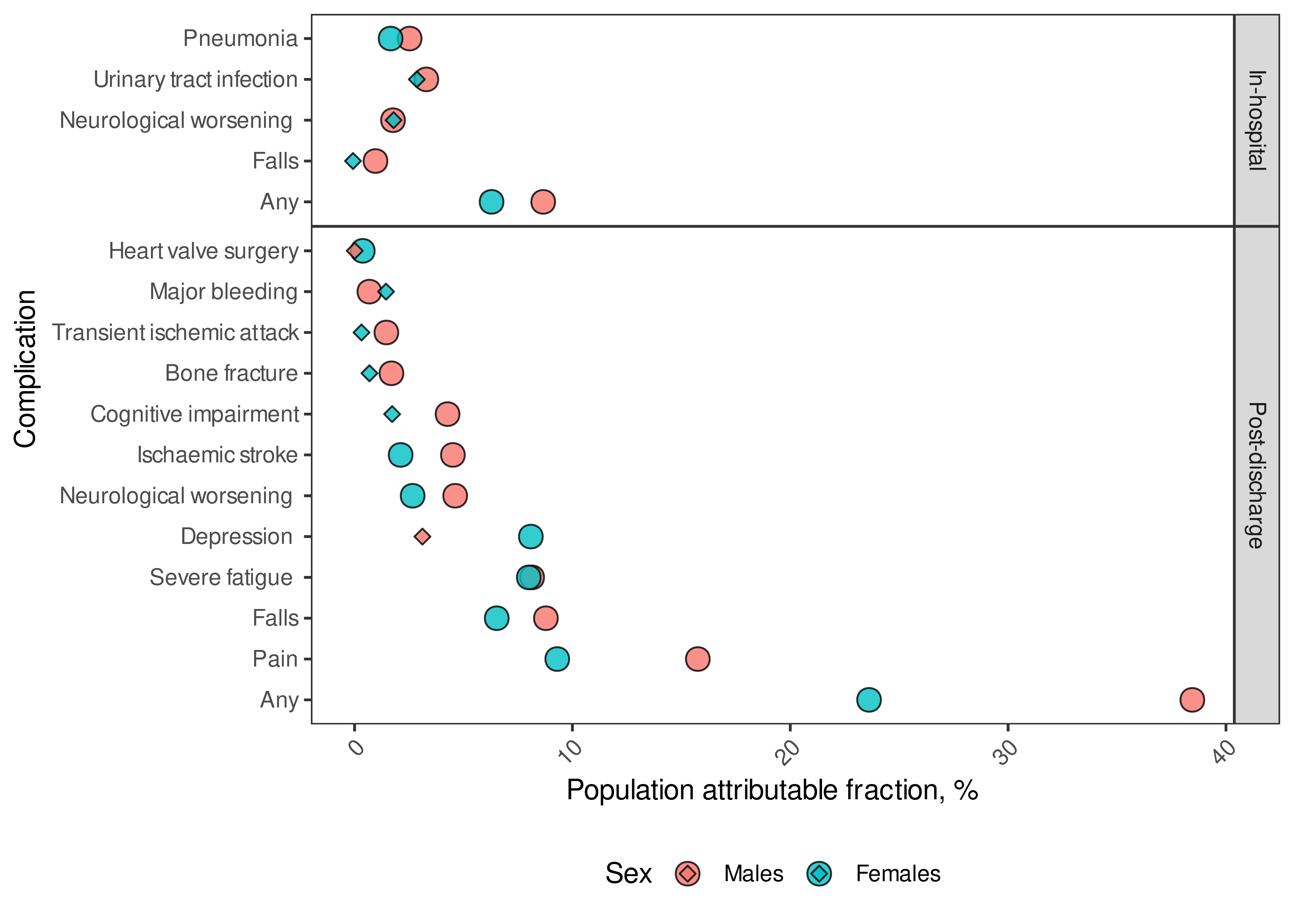


***Supplemental Figure 2d*** *- Impact of post-stroke complications in subgroups by prestroke functional status.*

*
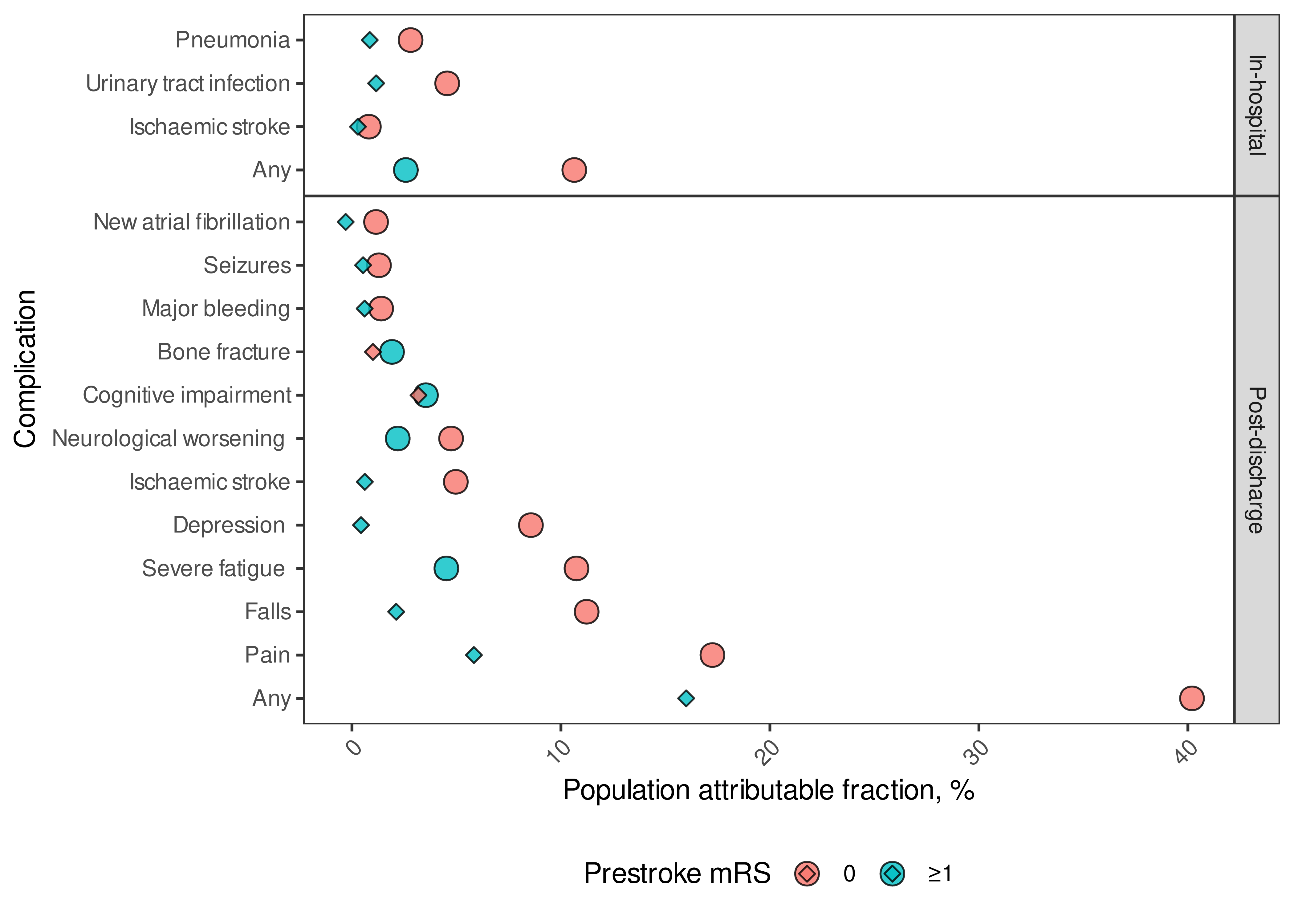
*

***Supplemental Figure 2e*** *- Impact of post-stroke complications in subgroups by catchment area.*

**
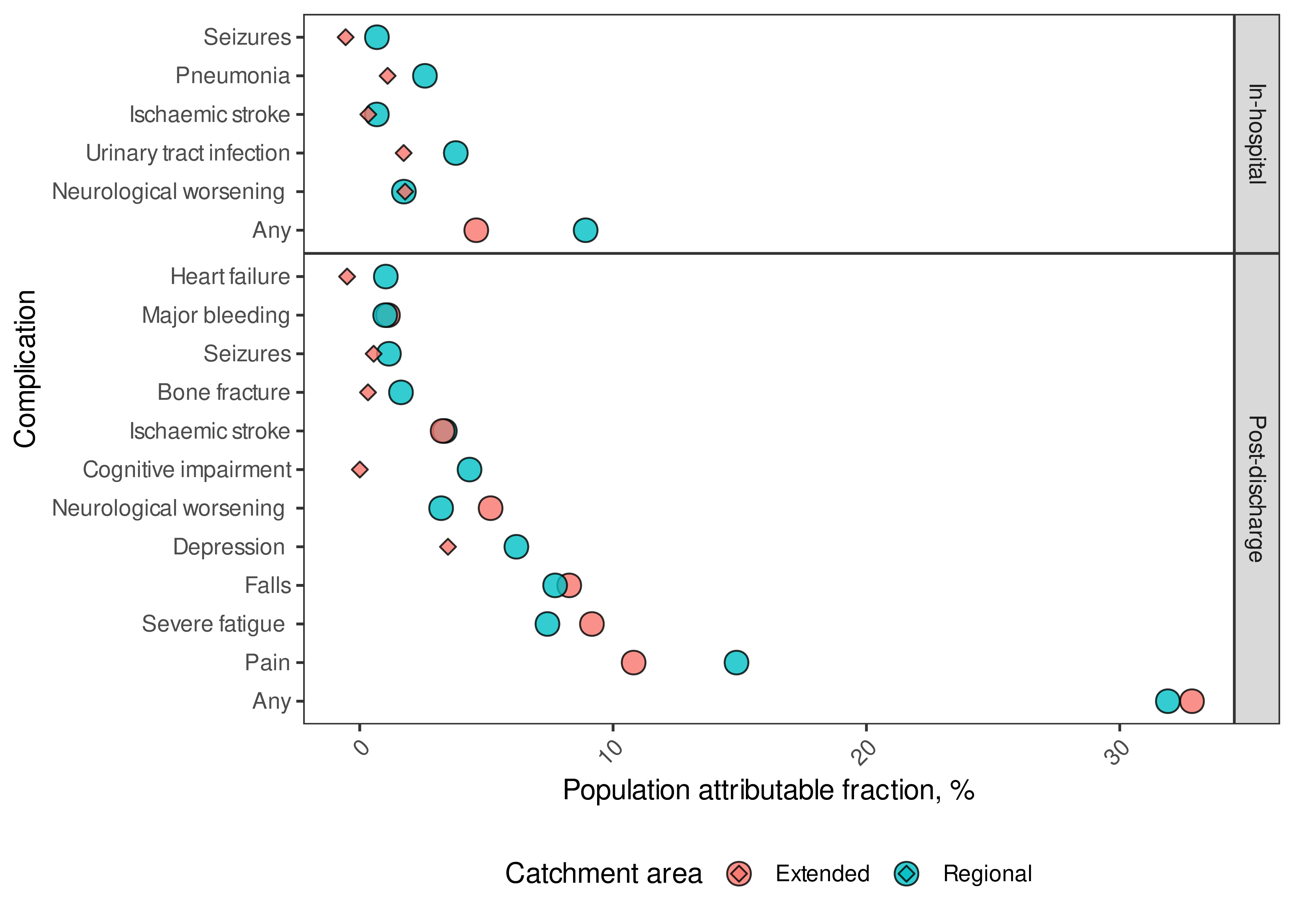
**

## **Supplemental Figure 3.** Impact of post-stroke complications on secondary endpoints.


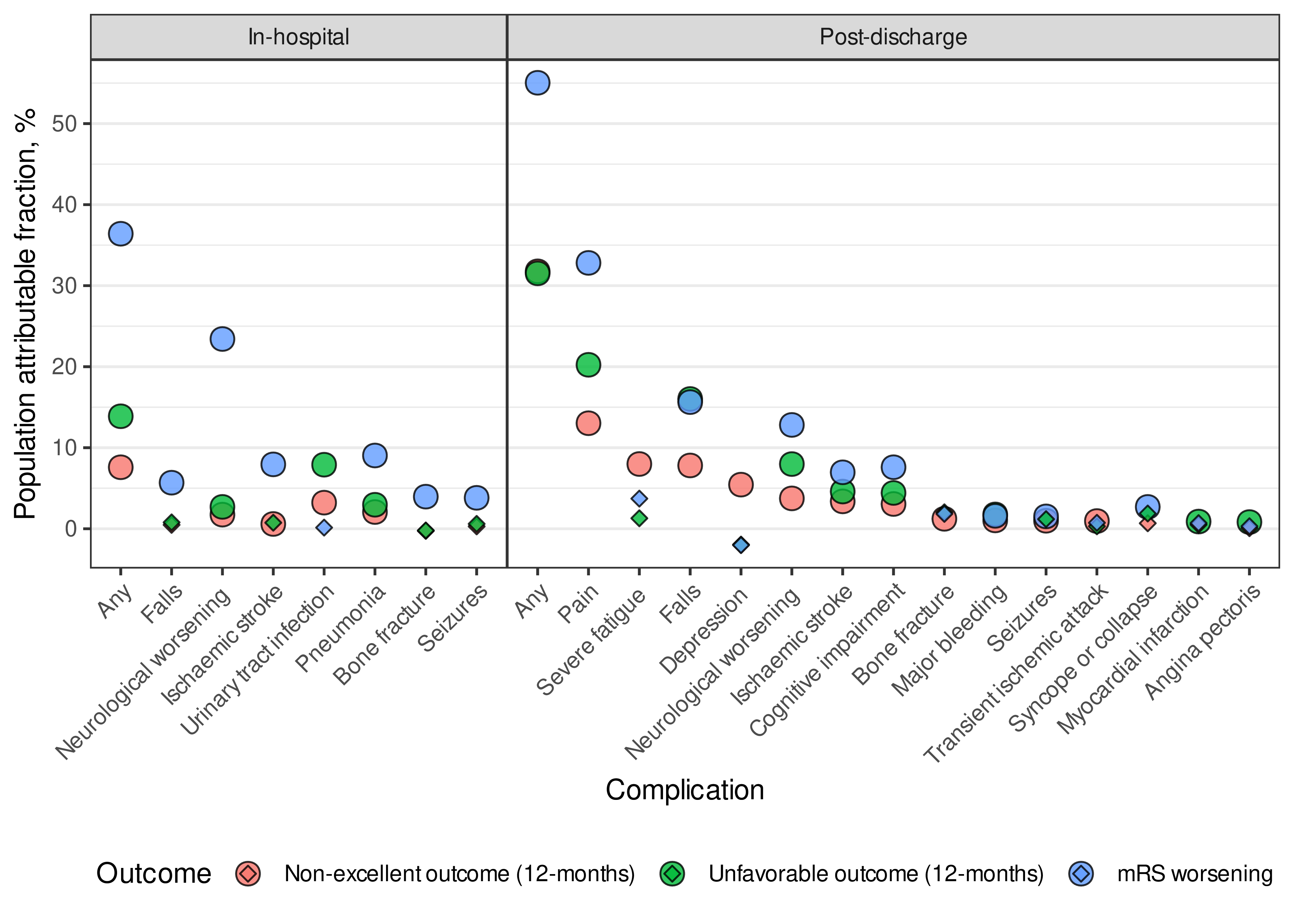
Population attributable fractions are shown for the complications significantly associated with the primary endpoint of non-excellent outcome (mRS>1), the secondary endpoint of unfavorable outcome (mRS>2), or the secondary endpoints of mRS worsening. Points indicate significant associations, diamonds non-significant associations.

Fewer complications were associated with unfavorable outcome than with non-excellent outcome. mRS worsening reflects any increase in mRS during each observation period and is the most sensitive end-point. Correspondingly, in-hospital falls, fractures, and seizures, and post-discharge syncope were only associated with mRS worsening.

mRS, modified Rankin Scale.

# References

1. Sacco RL, Kasner SE, Broderick JP, et al. An updated definition of stroke for the 21st century: a statement for healthcare professionals from the American Heart Association/American Stroke Association. *Stroke J Cereb Circ* 2013; 44: 2064–2089.

2. Thygesen K, Alpert JS, Jaffe AS, et al. Fourth Universal Definition of Myocardial Infarction (2018). *Glob Heart* 2018; 13: 305–338.

3. Knuuti J, Wijns W, Saraste A, et al. 2019 ESC Guidelines for the diagnosis and management of chronic coronary syndromes: The Task Force for the diagnosis and management of chronic coronary syndromes of the European Society of Cardiology (ESC). *Eur Heart J* 2020; 41: 407–477.

4. McDonagh TA, Metra M, Adamo M, et al. 2021 ESC Guidelines for the diagnosis and treatment of acute and chronic heart failure: Developed by the Task Force for the diagnosis and treatment of acute and chronic heart failure of the European Society of Cardiology (ESC) With the special contribution of the Heart Failure Association (HFA) of the ESC. *Eur Heart J* 2021; 42: 3599–3726.

5. Lim W, Le Gal G, Bates SM, et al. American Society of Hematology 2018 guidelines for management of venous thromboembolism: diagnosis of venous thromboembolism. *Blood Adv* 2018; 2: 3226–3256.

6. Schulman S, Kearon C, Subcommittee on Control of Anticoagulation of the Scientific and Standardization Committee of the International Society on Thrombosis and Haemostasis. Definition of major bleeding in clinical investigations of antihemostatic medicinal products in non-surgical patients. *J Thromb Haemost JTH* 2005; 3: 692–694.

7. Brignole M, Moya A, de Lange FJ, et al. 2018 ESC Guidelines for the diagnosis and management of syncope. *Eur Heart J* 2018; 39: 1883–1948.

8. Anderson JL, Halperin JL, Albert NM, et al. Management of patients with peripheral artery disease (compilation of 2005 and 2011 ACCF/AHA guideline recommendations): a report of the American College of Cardiology Foundation/American Heart Association Task Force on Practice Guidelines. *Circulation* 2013; 127: 1425–1443.
